# Effects of fertilizer reduction coupled with straw returning on soil fertility, wheat root endophytic bacteria, and the occurrence of wheat crown rot

**DOI:** 10.3389/fmicb.2023.1143480

**Published:** 2023-03-31

**Authors:** Yajiao Wang, Yuxing Wu, Caiyun Cao, Sen Han, Weisong Zhao, Qiusheng Li, Xuetong Liu, Lingxiao Kong

**Affiliations:** ^1^Institute of Plant Protection, Hebei Academy of Agricultural and Forestry Sciences, Baoding, China; ^2^Institute of Dryland Farming, Hebei Academy of Agricultural and Forestry Sciences, Hengshui, China

**Keywords:** excessive fertilization, fertilizer reduction, straw returning, endophytic bacteria, wheat crown rot

## Abstract

Excessive fertilization is associated with nutrient loss, soil compaction, and weak plant resistance. Straw returning can increase soil fertility with a consequent reduction in fertilizer, but the effects of fertilizer reduction coupled with straw returning on crop endophytic microbes and crop disease are poorly understood. Therefore, using metagenomic sequencing methods we investigated the responses of soil fertility, diversity, the function of root endophytic bacteria, and the occurrence of wheat crown rot due to the application of fertilizer (no, moderate and excessive fertilizer) coupled with or without straw returning after 7 years of treatments. The results showed that, after excessive fertilization, the wheat crown rot became severe, registering a disease index of 23. Compared with excessive fertilization, moderate fertilization coupled with straw returning significantly reduced the incidence of wheat crown rot, the disease index was reduced by 38.50%, and the richness and diversity of endophytic bacteria were increased by 61.20 and 11.93%, respectively, but the soil fertility was not significantly affected. In addition, moderate fertilization coupled with straw returning changed the community structure of endophytic bacteria and increased the relative abundance of carbohydrate metabolism and nitrogen fixation-related genes by 4.72 and 9.32%, respectively. Our results indicated that fertilizer reduction coupled with straw returning reduced the occurrence of wheat crown rot, increased the diversity of endophytic bacteria, and changed the community structure and function of endophytic bacteria, which will provide a better understanding of the interaction of fertilization coupled with straw returning, endophytic bacteria and wheat crown rot.

## 1. Introduction

China is a largely agricultural country, producing 30% of grains on 7% of the world's cultivated lands (Hou et al., 2021). To increase grain yield per unit area, large amounts of fertilizer are used to increase soil fertility. In 2021, 51.91 million tons of chemical fertilizer were applied in China, accounting for 30% of global consumption, and the unit area consumption reached 362 kg/hm^2^, which is 3.7 times the world average level (National Bureau of Statistics, https://data.stats.gov.cn/). As an important nutrient source for crop development and growth, fertilizer plays an important role in increasing crop yield. However, excess application of fertilizer not only causes soil compaction and nutritional imbalance but also weakens plant resistance to diseases and insects (Li et al., 2021). As straw is rich in organic matter and nutrients such as nitrogen, phosphorus, and potassium, straw returning is considered effective field management to increase soil fertility and reduce the use of chemical fertilizer (Wang Z. et al., 2021). In addition, straw returning can also promote the formation of soil aggregate structure, enhance soil permeability, reduce soil bulk density, and alleviate soil compaction and degradation due to excess fertilizer application (Xu et al., 2019; Yang and Lu, 2022). However, residual pathogens and pests' eggs in the straw may increase if it is mulched back into the soil (Wang, 2012). Therefore, to increase soil fertility and ensure the healthy growth of crops, balancing the amount of fertilizer and straw mulching are vital.

Endophytes are microbes that spend all or part of their lives in plant tissues which are strongly associated with plant growth and health (Brader et al., 2014; Salvi et al., 2022). Endophytes promote nutrient uptake, enhancing the growth of host plants and improving their resistance to insect pests and diseases, high temperature, drought, salinity, and alkalinity (Hardoim et al., 2015; Khare et al., 2018; Papik et al., 2020). Endophytes can protect plants from invading pathogens in three ways (Žiarovská et al., 2020). The first way is direct effect inhibition, in which endophytes can directly inhibit or kill pathogenic bacteria by secreting antibiotic substances (antibacterial, antifungal secondary metabolites, or lytic enzymes). The second way is indirect inhibition, in which inherent plant defense [systemic acquired resistance (SAR) and induced systemic resistance (ISR)] pathways can be elicited by endophytes (Xu et al., 2021). For example, *Pseudomonas* and *Bacillus*, which are widespread in plants, can induce systemic resistance in plants and lead to a higher tolerance through antibiotics, siderophores, volatiles, and other secondary metabolites (Van Loon et al., 2008; Hajabdollahi et al., 2021). The third way of pathogen suppression is ecological niche occupation. Endophytic *Methylobacterium* spp. inhibited the occurrence of citrus variegated chlorosis by occupying the same ecological niche of pathogen *Xylella fastidiosa* in citrus (Marcon et al., 2002). Endophytic bacteria are easily affected by field management (such as irrigation, fertilization, and pesticides), Therefore, studying the effects of field management on community structure and the function of endophytes plays an important role in crop health management.

Wheat crown rot is a widespread disease throughout the world and is predominantly caused by *Fusarium pseudograminearum* in China (Ji et al., 2016; Rahman et al., 2021). *F. pseudograminearum* colonizes the wheat stalks, blocking the transport of water and nutrients and eventually causing whiteheads before maturity and reducing yield (Smiley, 2019; Ozdemir et al., 2020). Wheat crown rot occurred relatively late in China but has dramatically increased in recent years due to the application of excess fertilizers and straw returning. However, research has found that overapplication of nitrogenous fertilizer leads to preanthesis biomass production, leading to early stored soil water depletion, thereby aggravating the occurrence of wheat crown rot (Davis et al., 2009; Baha Eddine et al., 2019). As pathogens survive in the residues of wheat, straw returning increases the preliminary infection source and promotes the occurrence of wheat crown rot (Backhouse, 2006). Although some studies have examined the effects of fertilization, straw returning on wheat crown rot occurrence and their relationship with endophytes under different amounts of fertilizer application coupled with straw returning are poorly understood.

Since the North China plain is one of the primary grain-producing areas with a winter wheat–summer maize cropping system, the sustainable utilization of agricultural soil in this major grain-producing region could affect China's food security (Wang Y. et al., 2021; Wang Z. et al., 2021). In recent decades, soil compaction and nutrient imbalance occurred in the area due to excessive fertilization, and straw returning is widely used to increase soil fertility and reduce fertilizer application (Gao et al., 2011). However, the effects of fertilizer reduction coupled with straw returning on crop endophytic microbes and crop disease are poorly understood. In this study, we investigated the soil chemical properties, endophytic bacteria diversity, and the occurrence of wheat crown rot after 7 years of treatments of the application of different amounts of fertilizer coupled with or without straw returning. The objectives of the study were to (1) determine the effects of different fertilizer levels of excessive fertilization on endophytic bacteria diversity and the occurrence of wheat crown rot; (2) assess the effects of straw returning under different fertilizer conditions on soil properties, root endophytic bacteria, and the occurrence of wheat crown rot; (3) choose the optimum amount of fertilizer coupled with straw returning to reduce fertilizer application; and (4) determine the effects of fertilizer reduction coupled with straw returning on wheat root endophytic bacteria and the occurrence of wheat crown rot.

## 2. Materials and methods

### 2.1. Site description and sample collection

A long-term field experiment was carried out in 2013 at Shenzhou county (37°41′ N, 116°15′ E, 18 m a.s.l.), Hebei Province, China, which has a subtropical monsoon climate. The annual temperature is ~12.6°C with an annual precipitation of ~522 mm. Winter wheat (var. Jimai 22) is rotated with summer maize (var. Zhengdan 958). The experiment had six treatments that combined fertilizer application and crop straw returning practices as follows: (1) F1_NS: Before sowing wheat and corn, crop straws were removed and no chemical fertilizer was applied; (2) F1_S: Before sowing wheat and corn, crop straws were mulched back into the field and no chemical fertilizer was applied; (3) F2_NS: Before sowing wheat and corn, crop straws were removed and moderate chemical fertilizer [nitrogen (N) fertilizer, 180 kg/ha; phosphorus (P) fertilizer, 120 kg/ha; and potassium (K) fertilizer 120 kg/ha] was applied; (4) F2_S: Before sowing wheat and corn, crop straws were mulched back into the field and moderate chemical fertilizer (N fertilizer, 180 kg/ha; P fertilizer, 120 kg/ha; and K fertilizer 120 kg/ha) was applied; (5) F3_NS: Before sowing wheat and corn, crop straws were removed and excessive chemical fertilizer (N fertilizer, 540 Kg/ha; P fertilizer, 360 kg/ha; K fertilizer, 360 kg/ha) was applied; (6) F3_S: Before sowing wheat and corn, crop straws were mulched back into the field and excessive chemical fertilizer (N fertilizer 540 kg/ha; P fertilizer, 360 kg/ha; K fertilizer, 360 kg/ha) was applied (Supplementary Table S1). Regarding straw returning, maize residues were chopped into pieces (5 cm length) and incorporated into the soils when the field was plowed before winter wheat sowing, while wheat residues were chopped into pieces and left on the soil surface as mulch after harvest (Wang et al., 2019). The treatments were applied to 40 m^2^ (5 m × 8 m) plots arranged in a sequential block design, with four replicates per treatment. The same treatment was applied to each plot for 7 years using local farming practices for cultivation and field management (Yao et al., 2017).

Root samples were collected on 15 May 2020 (wheat-filling stage). Samples were collected according to the “W” multipoint sampling method, 10 sites were collected at each treatment, and 10 wheat plants were collected at each site. The fresh wheat roots were gently shaken to remove the surface soil and subsequently placed in sterilized phosphate-buffered saline (PBS) and shaken for 30 min before the rhizosphere soil was collected for centrifugation at 12,000 *g* for 5 min. The collected rhizosphere soil was air-dried under ambient temperature and stored at 4°C for soil analysis. Roots without rhizosphere soil were surface sterilized with 75% alcohol for 3 min, sodium hypochlorite solution (3% available chlorine) for 5 min, and rinsed 5 times with sterile water. The surface-sterilized roots were cut into 3-mm pieces and stored in liquid nitrogen for further analysis (Li et al., 2019).

Microbial genomic DNA was extracted from 0.5 g of root sample with the QuickExtract Plant DNA Extraction Kit (Omega Bio-TEK, Norcross, GA, USA) according to the manufacturer's instructions. The DNA quality was assessed using 1% agarose gel electrophoresis, and the concentration and purity were determined using a NanoDrop 2000 ultraviolet–visible (UV-Vis) spectrophotometer (Thermo Scientific, Wilmington, DE, USA).

### 2.2. Soil chemical property analysis

Rhizosphere soil samples were ground and sieved before soil analysis. Soil organic carbon (SOC) was measured by the chromic acid titration method; total nitrogen (TN) was determined using the alkali hydrolyzation procedure; available phosphorus (AP) was determined using the sodium hydrogen carbonate solution–Mo–Sb antispectrophotometric method. Available potassium (AK) was measured by the ammonium acetate method, followed by flame photometric detection (Wang Y. et al., 2021).

### 2.3. Quantitative detection of *F. pseudograminearum* in roots and evaluation of the occurrence of wheat crown rot

The q-PCR analysis for *F. pseudograminearum* titers in wheat root was performed with primers Fptri3eF (5′-CAAGTTTGATCCAGGGTAATCC-3′), Fptri3eR (5′-GCTGTTTCTCTTAGTCTTCCTCA-3′) (Obanor and Chakraborty, 2014). Additionally, 1 μl of template DNA was used in a 20 μl q-PCR reaction volume, and the reaction mix was according to the TaKaRa Ex Taq HS DNA Polymerase manufacturer's instructions. The amplification protocol was 95°C for 30 s, followed by 40 cycles at 95°C for 5 s and 60°C for 34 s on an ABI 7500 Real-Time PCR system. All reactions were performed in triplicate. The number of *F. pseudograminearum* genomes in a reaction was calculated using a recombinant plasmid standard curve containing the target sequence.

The severity of wheat crown rot was scored on a scale from 0 to 4 based on the percentage of crown area with necrosis lesions: 0 = completely healthy; 1 = <25% necrosis; 2 = 25–50% necrosis; 3 = 51–75% necrosis; 4 = >75% necrosis (Wildermuth, 1994). A disease index was then calculated as ∑ (Number of diseased plants with each score × Highest score)/(Total number of plants × Highest score) × 100%.

### 2.4. Illumina MiSeq sequencing and processing of sequencing data

The V5–V7 regions of the bacteria were amplified and underwent two rounds of amplification. The first round of PCR reactions was conducted using primers 799 F (5′-AACMGGATTAGATACCCKG-3′) and 1392 R (5′-ACGGGCGGTGTGTRC-3′), barcode was added before the primer to build different libraries in the same sequencing pool. The PCR mixture contained 5× TransStart FastPfu buffer of 4 μl; 2.5-mM dNTPs of 2 μl; forward primer (5 μM) of 0.8 μl; reverse primer (5 μM) of 0.8 μl; TransStart FastPfu DNA Polymerase of 0.4 μl; template DNA of 10 ng, with ddH_2_O added to reach 20 μl. The thermocycling conditions for the PCR in an ABI GeneAmp 9700 PCR thermocycler (ABI, CA, USA) were initial denaturation at 95°C for 3 min; 27 cycles of denaturation at 95°C for 30 s, annealing at 55°C for 30 s, and extension at 72 °C for 45 s; and single extension at 72°C for 10 min, and ends at 4°C. Subsequently, for each reaction, the PCR product was used as a template for a nested PCR amplification with primers 799F (5′-AACMGGATTAGATACCCKG-3′) and 1193 R (5′-ACGTCATCCCCACCTTCC-3′). The nested PCR was performed using a touchdown PCR program: initial denaturation at 95°C for 3 min, 13 cycles of denaturing at 95°C for 30 s, annealing at 55°C for 30 s, and extension at 72°C for 45 s; and single extension at 72°C for 10 min, and ends at 4°C (Liu et al., 2014). The PCR reactions were performed in triplicate. The PCR product was extracted from 1.5% agarose gel and purified using the AxyPrep DNA Gel Extraction Kit (Axygen Biosciences, Union City, CA, USA) according to the manufacturer's instructions and then quantified using a Quantus Fluorometer (Promega, Madison, WI, USA).

Purified amplicons were paired-end sequenced (2 × 300) on an Illumina MiSeq platform (Illumina, San Diego, CA, USA) according to standard protocols by Majorbio Bio-Pharm Technology Co. Ltd. (Shanghai, China). The raw reads were deposited in the NCBI Sequence Read Archive (SRA) database (Accession No. PRJNA743589).

The raw sequencing reads were demultiplexed, quality filtered by Trimmomatic, and merged by FLASH (Magoc and Salzberg, 2011). Operational taxonomic units (OTUs) with a 97% similarity cutoff were clustered using UPARSE (version 7.1, http://drive5.com/uparse/), and chimeric sequences were identified and removed. The OTU abundances were rarefied to the lowest number of shared OTUs to remove the effect of sequencing depth across samples. The taxonomy of each gene sequence was analyzed using the SILVA reference gene database (http://www.arb-silva.de/) with a confidence threshold of 70% (Kõljalg et al., 2013). The OTUs affiliated with chloroplasts and mitochondria were removed.

### 2.5. Statistical analyses

The alpha diversity of the bacterial community (including Shannon and Simpson indices) was calculated by Mothur version v.1.30 (http://www.mothur.org/) in R with the vegan package. Principal coordinate analysis (PCoA) analysis was performed in R using Bray–Curtis distances, which was used to compare the beta diversity of the bacterial community. Analysis of similarities (ANOSIM) was carried out to test for significant differences in fungal communities of different cropping systems. ANOSIM was performed in R using the vegan package with the Bray–Curtis distance and 999 permutations. Redundancy analysis (RDA) was executed in R version 3.0.2 to analyze the relationship between the dominant OTU of endophytic bacteria and the environmental factors. Correlation networks were used to examine the interaction among environmental factors, bacterial diversity indices, and wheat crown rot conducted in R with igraph and psych packages. To simplify the networks for better visualization only the 50 most abundant genera were analyzed and strong interactions with a *p*-value of <0.05 and Spearman's *p*-value of ≥0.50 were examined. The cutoff value for the similarity matrix was 95%, and the visualization of the network was performed in Gephi (version 0.9.2; https://gephi.org/) with a Fruchterman–Reingold layout algorithm. All data were checked for normality by the Shapiro–Wilk test before statistical analysis by SPSS version 16.0 (SPSS, Chicago, IL, USA). Alpha diversity indices, wheat crown rot, and relative abundance of the major genus, which were non-normally distributed, were tested for significant differences using non-parametric statistics and the Kruskal–Wallis test using SPSS version 16.0.

## 3. Results

### 3.1. Soil properties

The effects of fertilization coupled with straw returning on soil chemical properties are presented in Table 1. The contents of SOC, TN, AP, and AK increased significantly with the increase in fertilizer application. Compared with no fertilizer (F1-NS), the contents of SOC, TN, AP, and AK increased by 80.33, 32.07, 33.12, and 65.31%, respectively, after medium fertilization (F2-NS) and increased by 139.45, 122.90, 45.36, and 104.04%, respectively, after excessive fertilization (F3-NS). Straw returning significantly increased the content of SOC, TN, AP, and AK under the condition of no fertilization and medium fertilization but had no significant effect under the condition of excessive fertilization. Compared with straw removal, the content of SOC, TN, AP, and AK increased by 42.69, 13.06, 51.62, and 22.36%, respectively, under medium fertilization and increased by 25.50, 55.25, 17.51, and 20.07%, respectively, under no fertilization. In addition, no significant difference was present between the SOC, TN, AP, and AK contents under moderate fertilization coupled with straw returning (F2-S) and under excessive fertilization coupled with straw removal (F3-NS). This result indicated that straw returning after excessive fertilization can minimize fertilizer use without reducing soil fertility.

**Table 1:** Means ± standard deviation for soil chemical properties variables.

**Treatments**	**SOC g/kg**	**TN g/kg**	**AP mg/kg**	**AK mg/kg**
F1-NS	13.33 ± 1.49d	0.64 ± 0.05c	21.42 ± 2.35c	145.34 ± 8.48d
F1-S	19.02 ± 0.95c	0.72 ± 0.08bc	32.47 ± 1.48a	177.84 ± 10.32c
F2-NS	24.04 ± 0.87b	0.85 ± 0.06b	28.51 ± 1.25b	239.69 ± 18.64b
F2-S	30.17 ± 1.25a	1.32 ± 0.13a	32.33 ± 1.35a	287.81 ± 20.85a
F3-NS	31.92 ± 0.76a	1.43 ± 0.07a	31.14 ± 0.58a	295.86 ± 22.65a
F3-S	32.67 ± 1.85a	1.55 ± 0.18a	32.39 ± 2.45a	300.93 ± 25.45a

Different lowercase letters within a column indicate a significant difference in a variable among sites (p < 0.05) according to Tukey's test. F1_S, straw returning with no chemical fertilizer application; F2_NS, non-straw returning with moderate chemical fertilizer application; F2_S, straw returning with moderate chemical fertilizer application; F3_NS, non-straw returning with excessive chemical fertilizer application; F3_S, straw returning with excessive chemical fertilizer application; SOC, soil organic carbon; TN, total nitrogen; AK, available potassium, AP, available phosphorus.

### 3.2. Effects of straw returning coupled with fertilization on the population of *F. pseudograminearum* in wheat root and the occurrence of wheat crown rot

**Figure 1:**
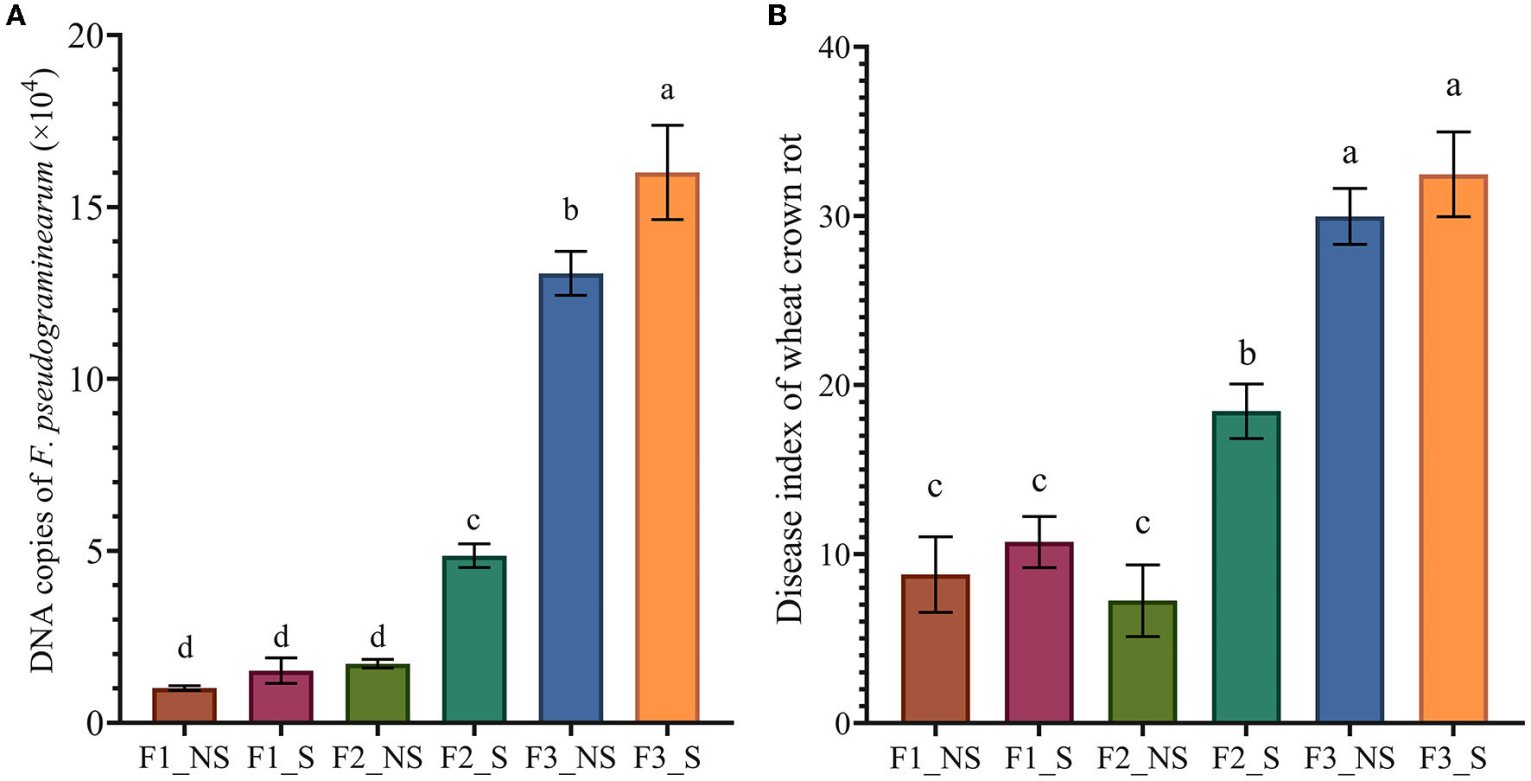
The abundance of *F. pseudograminearum* and disease index of wheat crown rot in different treatments. **(A)** Quantification of *F. pseudograminearum* in the wheat root by real-time PCR; **(B)** Disease index of wheat crown rot. Data for the same disease with different letters above bars differed significantly (*p* < 0.05) according to Tukey's test. F1_NS, non-straw returning with no chemical fertilizer application; F1_S, straw returning with no chemical fertilizer application; F2_NS, non-straw returning with moderate chemical fertilizer application; F2_S, straw returning with moderate chemical fertilizer application; F3_NS, non-straw returning with excessive chemical fertilizer application; F3_S, straw returning with excessive chemical fertilizer application.

The effects of straw returning coupled with fertilization on the population of *F. pseudograminearum* in the wheat root were determined by real-time PCR (Figure 1A). The results showed that compared with no fertilizer application, the population of *F. pseudograminearum* increased with the increase in fertilizer application. Without straw returning, the populations of *F. pseudograminearum* in F2_NS and F3_NS were 1.7 and 13.07 times greater than that in F1_NS, respectively, while with straw returning, the populations of *F. pseudograminearum* in F2_S and F3_S were 3.86 and 10.53 times greater than those in F1_S, respectively. Furthermore, the results showed that, after straw returning, the population of *F. pseudograminearum* increased by 51.97, 713.89, and 22.14% in F1_S (compared with F1_NS), F2_S (compared with F2_NS), and F3_S (compared with F3_NS), respectively.

The disease index of wheat crown rot was examined (Figure 1B), and the results showed that excessive fertilization significantly increased the disease index of wheat crown rot. The disease index was 30 in F3_NS, which was 214.30 and 313.79% higher than that in F1_NS and F2_NS, respectively. Similarly, the disease index was 32.45 in F3_S, which was 202.71 and 75.88% higher than that in F1_NS and F2_NS, respectively. After straw returning, the disease index of wheat crown rot increased by 21.82, 154.48, and 8.2% in F1_S (compared with F1_NS), F2_S (compared with F2_NS), and F3_S (compared with F3_NS), respectively. In addition, fertilizer reduction coupled with straw returning significantly decreased the disease index of wheat crown rot. The disease index was 18.45 in F2_S, which was 38.50% lower than that in F3_NS. The results showed that both excess fertilization and straw returning could increase the occurrence of wheat crown rot, but the increased effect due to excess fertilization was greater. Reducing fertilizer application could significantly reduce the incidence of wheat crown rot.

### 3.3. Endophytic bacterial diversity

After filtering the low-quality reads, removing the barcodes and primers, and trimming chimeras, 1,519,465 high-quality sequences were obtained across all samples. The number of reads per sample ranged from 51,752 to 74,433. Each sample was normalized to 51,752 reads to conduct downstream analyses with an average length of 377 bp based on a 97% OTUs similarity threshold (Supplementary Table S2). A total number of 1,456 OTUs were obtained from root endophytes, including 25 phyla, 57 classes, 137 orders, 240 families, and 445 genera. The coverage of all samples was above 99.55% (Supplementary Table S1), which indicated that most of the endophytic bacteria had been detected, and the sequencing library reached saturation. Thus, the results validly represented the sample condition.

**Figure 2:**
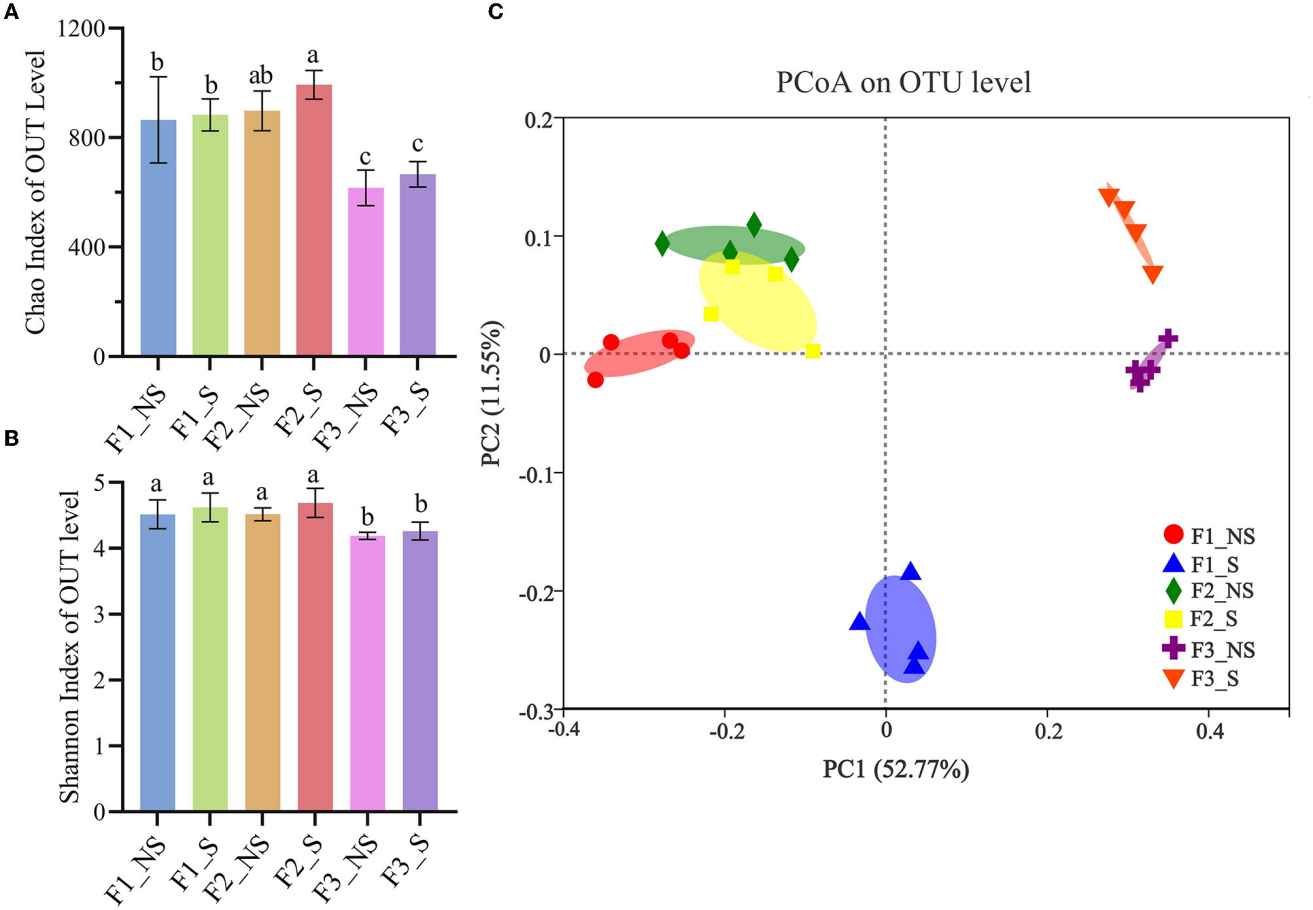
Alpha and beta diversity of endophytic bacteria in wheat root after fertilizer application and straw returning. Variation in the alpha diversity of endophytic bacteria based on the Shannon index **(A)** and Chao index **(B)**. Data for the same index with different letters above bars differed significantly (*p* < 0.05) according to Tukey's test. Principal coordinate analysis of endophytic bacteria **(C)** based on Bray–Curtis distance matrices. The *x*- and *y*-axis represent the two selected principal coordinate axes; the percentage represents the explanatory value of the principal coordinate axes for the difference in sample composition; distances between symbols on the ordination plot reflect relative dissimilarities. F1_NS, non-straw returning with no chemical fertilizer application; F1_S, straw returning with no chemical fertilizer application; F2_NS, non-straw returning with moderate chemical fertilizer application; F2_S, straw returning with moderate chemical fertilizer application; F3_NS, non-straw returning with excessive chemical fertilizer application; F3_S, straw returning with excessive chemical fertilizer application.

The Chao and Shannon indices for the richness and diversity, respectively, for endophytic bacteria, were calculated among treatments (Figures 2A, B). The Chao index in F2_NS was 898, which was 3.94% higher than that in F1_NS but the difference was not significant, and it was 45.78% higher than that in F3_NS. The Shannon indices in F1_NS and F2_NS were both 4.52, which was 6.10% higher than that in F3_NS. The results indicated that excess fertilization reduced the richness and diversity of endophytic bacteria in wheat roots. In F1_S, F2_S, and F3_S, the Chao indices were 2.20, 10.58, and 8.12% higher than those in F1_NS, F2_NS, and F3_NS, respectively. Similarly, the Shannon indices in F1_S, F2_S, and F3_S were 2.21, 3.76, and 1.67%, respectively, higher than those in F1_NS, F2_NS, and F3_NS, respectively, but all the differences were not significant. The results indicated that straw returning increased the richness and diversity of endophytic bacteria in wheat roots; however, the difference was not significant. The index of Chao and Shannon was 993 and 4.69, respectively, which was 61.20 and 11.93% higher than that in F3_NS. The results indicated that fertilizer reduction coupled with straw returning significantly increased the richness and diversity of endophytic bacteria in the wheat root.

The PCoA plot based on the OTU analyses showed that all treatments had a notable difference, and the first two principal coordinates explained 11.55 and 52.77% of the total variance of the root endophytic bacterial community structures (Figure 2C). F2_NS and F2_S had the same bacterial community structures on PCoA1 but had slightly different structures on PCoA2 as did F3_NS and F3_S. However, F1_NS and F1_S had different structures on both PCoA1 and PCoA2. The results indicated that straw returning had a greater effect on the endophytic bacterial structure under the low fertility status. Without straw returning, F1_NS and F2_NS had similar endophytic bacterial community structures but had significant differences with F3_NS. The results showed that excess fertilization significantly changed the endophytic bacterial community structure in the wheat root.

### 3.4. Endophytic bacterial community composition

**Figure 3:**
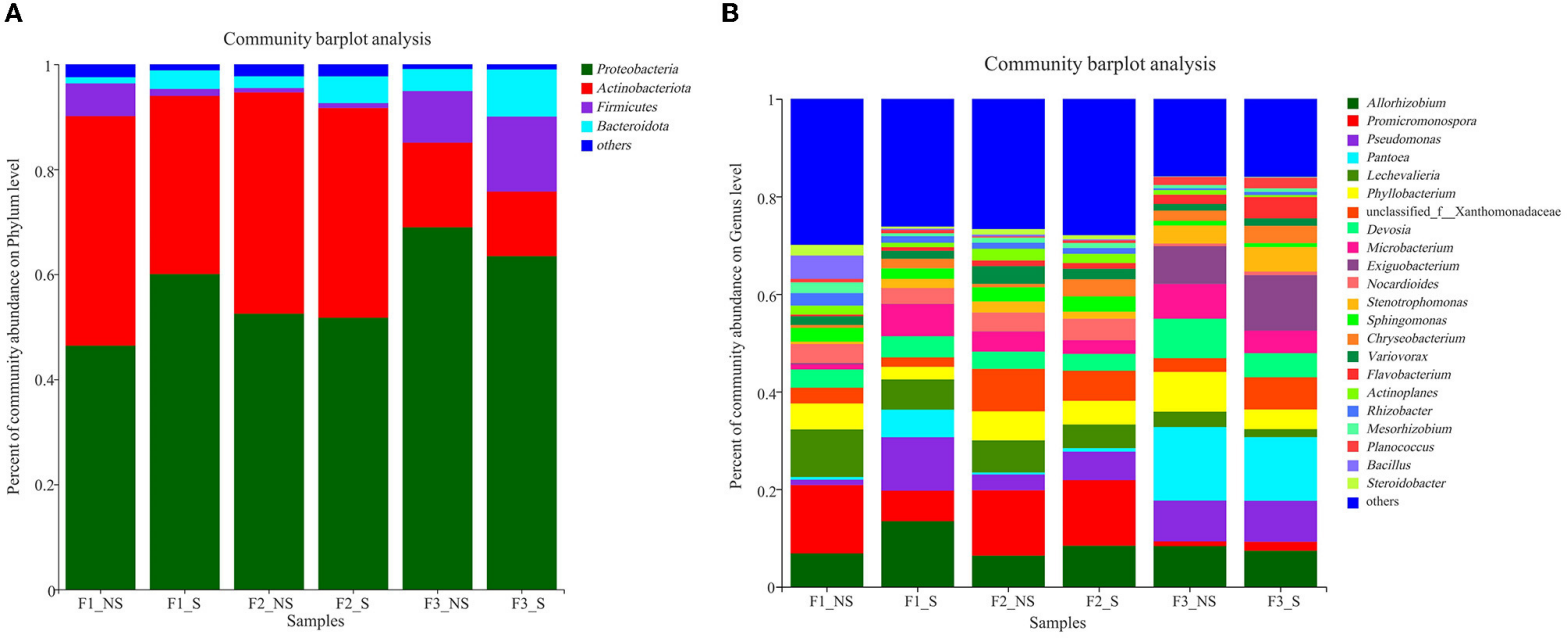
Relative abundances of endophytic bacterial phyla **(A)** and genera **(B)** in wheat root after fertilizer application and straw returning. F1_NS, non-straw returning with no chemical fertilizer application; F1_S, straw returning with no chemical fertilizer application; F2_NS, non-straw returning with moderate chemical fertilizer application; F2_S, straw returning with moderate chemical fertilizer application; F3_NS, non-straw returning with excessive chemical fertilizer application; F3_S, straw returning with excessive chemical fertilizer application.

The dominant phyla (relative abundance >1%) across all treatments were Proteobacteria, Actinobacteriota, Firmicutes, and Bacteroidota, accounting for more than 97% of the endophytic bacterial sequences (Figure 3A). After straw returning, the relative abundances of Actinobacteriota decreased by 28.82, 5.51, and 31.14%, respectively, under low (in F1_S compared with F1_NS), moderate (in F2_S compared with F2_NS), and excessive (in F3_S compared with F3_NS) fertilizer usages, but the relative abundances of Bacteroidota increased by 66.86, 56.30, and 53.33%, respectively. In F1_NS, F2_NS, and F3_NS (without straw returning), the relative abundances of Proteobacteria and Bacteroidota increased with the enhanced fertilization; however, the relative abundance of Firmicutes was the lowest in F2_NS.

**Figure 4:**
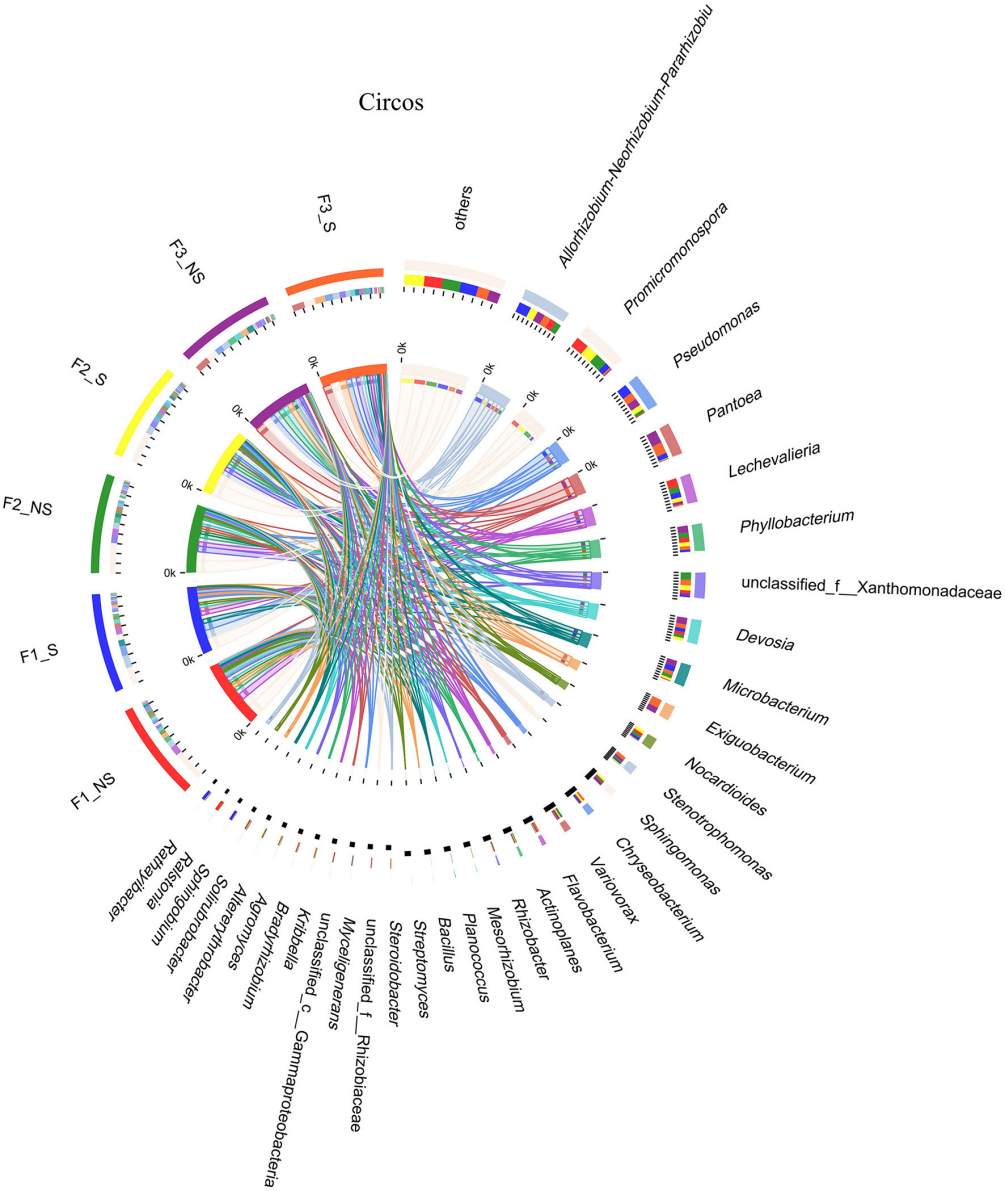
Circos analysis of distribution and proportion of endophytic bacteria in wheat root after fertilizer application and straw returning. In the left half of the circle, the axis represents the relative abundance of genera or function in the corresponding sample. In the right half of the circle, the axis represents the proportion of a certain genus or function in the cropping system in. F1_S, straw returning with no chemical fertilizer application; F2_NS, non-straw returning with moderate chemical fertilizer application; F2_S, straw returning with moderate chemical fertilizer application; F3_NS, non-straw returning with excessive chemical fertilizer application; F3_S, straw returning with excessive chemical fertilizer application.

The dominant genera with abundance >1% were analyzed (Figures 3B, 4). A total of 21 dominant genera were present in F1_NS, which shared 80.95 and 33.33% genera with F2_NS and F3_NS, respectively. The results indicated that excess fertilization significantly changed the composition of the dominant genera of endophytic bacteria in the wheat root. Among them, the relative abundances of *Promicromonospora, Lechevalieria, Bacillus, Rhizobacter, Mesorhizobium, Steroidobacter, Streptomyces, Ralstonia, Bradyrhizobium, Myceligenerans*, and *Kribbella* decreased with the increased fertilization (F1_NS > F2_NS > F3_NS), but the relative abundance of *Phyllobacterium* and *Pseudomonas* increased with the increased fertilizer application (F1_NS <F2_NS < F3_NS). After straw returning, F1_NS shared 61.90% dominant genera with F1_S but shared 89.47 and 100% genera with F2_S and F3_S, respectively (Supplementary Table S3). The results showed that, after straw returning, the composition of the dominant genera predominantly changed under low fertilization than under moderate or high fertilization.

### 3.5. Prediction of functional composition of the endophytic microbial community

**Figure 5:**
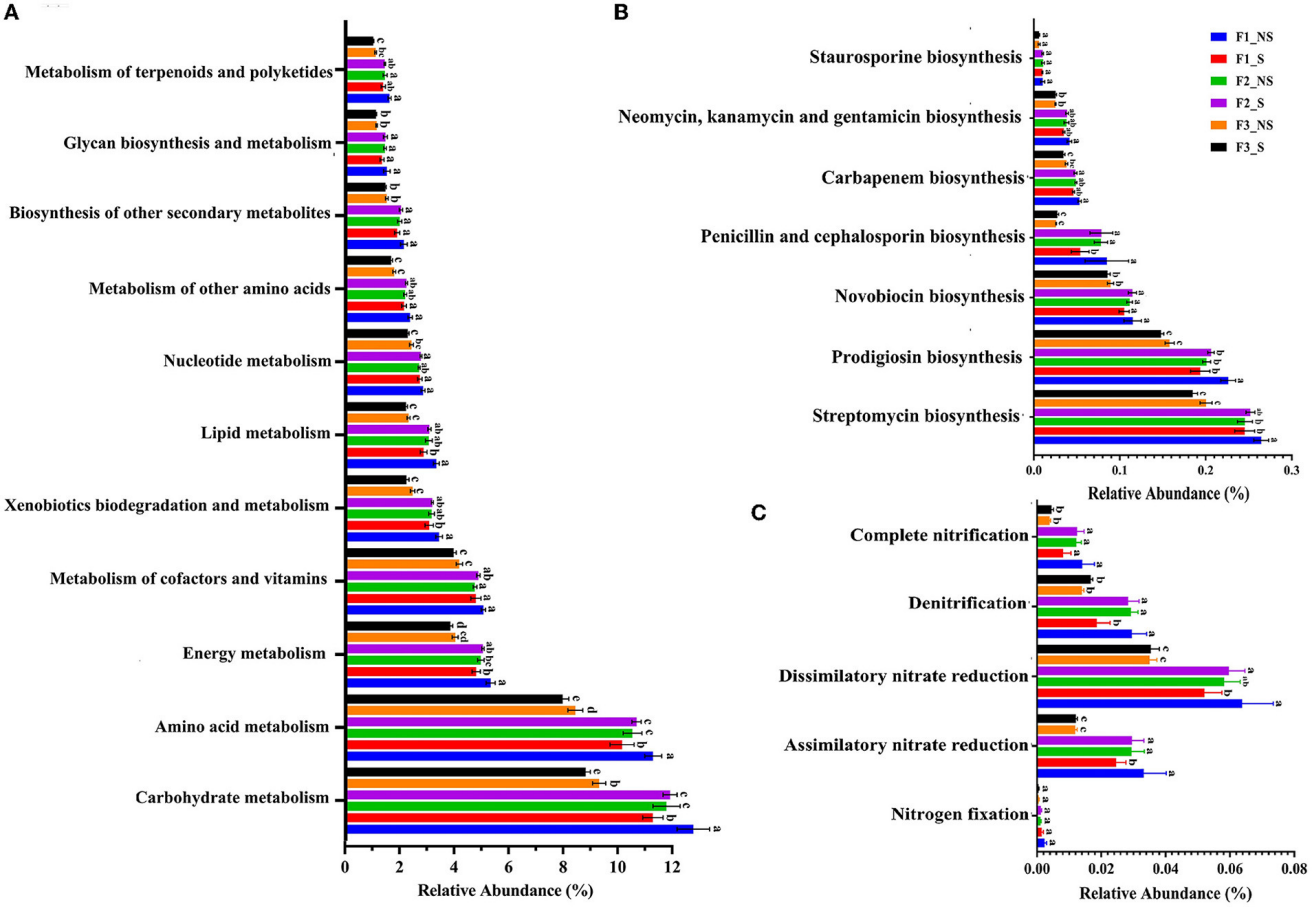
Function relative abundance of endophytic bacteria in wheat root after fertilizer application and straw returning. **(A)** Function relative abundance of endophytic bacteria at KEGG level 2. **(B)** Function relative abundance of antibiotic biosynthesis. **(C)** Function relative abundance of nitrogen metabolism. Data for the same index with different letters above bars differed significantly (*p* < 0.05) according to Tukey's test. F1_S, straw returning with no chemical fertilizer application; F2_NS, non-straw returning with moderate chemical fertilizer application; F2_S, straw returning with moderate chemical fertilizer application; F3_NS, non-straw returning with excessive chemical fertilizer application; F3_S, straw returning with excessive chemical fertilizer application.

The functional composition of the endophytic microbial community was predicted by PICRUSt2; it was found that the major functions were the metabolism pathway (75.27–77.91%), environmental information processing (6.66–7.71%), the genetic information process (5.32–5.58%), and the cellular processes (4.56–5.46%) in the level 1 of the Kyoto Encyclopedia of Genes and Genomes (KEGG) pathway. The metabolism process with the largest proportion contained 11 pathways (Figure 5A). Notably, the most abundant endophytic bacteria microbiota was related to carbohydrate metabolism, amino acid metabolism, and energy metabolism.

A vital role of endophytic bacteria is to help plants absorb nutrients. However, the relative proportion of carbohydrate metabolism pathway and nitrogen fixation decreased with the increased fertilizer application (F1_NS < F2_NS < F3_NS) (Figures 5B, C). After straw returning, the relative abundances of carbohydrate metabolism and nitrogen fixation-related genes decreased by 28.82 and 41.46%, respectively, in F1_S compared with F1_NS, but it had no significant change in F2_S and F3_S compared with F2_NS and F3_NS, respectively. Compared with F3-NS, the relative abundance of carbohydrate metabolism and nitrogen fixation-related genes increased by 4.72 and 9.32%, respectively, in F2-S. The results indicated that straw returning reduced the abundance of carbohydrate metabolism and nitrogen fixation-related genes in low fertility, but no significant difference was observed in medium and high fertilizer conditions. Fertilizer reduction coupled with straw returning significantly increased the abundance of carbohydrate metabolism and nitrogen fixation-related genes. There are seven types of antibiotic secondary metabolites produced by endophytic bacteria (Figure 5C). In F1_NS and F2_NS, the synthesis of penicillin and cephalosporin, carbapenem, neomycin kanamycin and gentamicin, and staurosporine had no difference but were significantly higher than those in F3_NS. After straw returning, the synthesis of the four types of antibiotics significantly decreased in F1_S compared with F1_NS, but they had no significant difference in F2_S and F3_S compared with F2_NS and F3_NS, respectively. Lipoarabinomannan is an important component of the bacterial cell membrane and can be used as an activator to induce systemic resistance in plants (Van Loon, 2007). Lipoarabinomannan biosynthesis significantly decreased with the fertilizer application and straw returning under low or excessive fertilization, but it had no significant change with straw returning under moderate fertilization (Supplementary Figure S1). The results indicated that the secretion of antibiotics by endophytic bacteria was reduced under excess fertilization but had no significant difference after straw returning in moderate or high fertility conditions. In general, excess fertilization changed the function of endophytic bacteria and decreased carbohydrate metabolism, nitrogen fixation, antibiotic, and Lipoarabinomannan synthesis, whereas straw returning under moderate fertilization had no significant effect on the function.

### 3.6. Correlation of environmental factors with endophytic bacterial community composition

**Figure 6:**
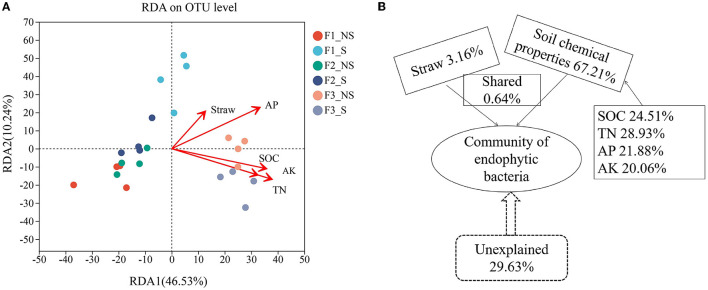
Correlation analysis of environmental factors with endophytic bacterial community compositions. Redundancy analysis showing the influence of environmental factors on the microbial community at the genus level **(A)**. Analysis of the contribution of environmental factors to changes in the endophytic bacterial community **(B)**. N, nitrogen fertilizer; P, phosphorus fertilizer; F1_S, straw returning with no chemical fertilizer application; F2_NS, non-straw returning with moderate chemical fertilizer application; F2_S, straw returning with moderate chemical fertilizer application; F3_NS, non-straw returning with excessive chemical fertilizer application; F3_S, straw returning with excessive chemical fertilizer application.

Redundancy analysis (RDA) was used to assess the effects of soil chemical properties and straw returning on the structure of endophytic bacterial communities (Figure 6A; Supplementary Table S4). The results revealed that total nitrogen was the most important factor (*p* < 0.05, *r*^2^ = 0.63). The variance partition analysis was used to dissect the contributions of the environmental factors (Figure 6B), and factors comprehensively accounted for 70.37% of the microbial community structural changes. Soil chemical properties contributed 67.21% of the variation, of which SOC, TN, AP, and AK contributed 24.51, 28.93%, 21.88, and 20.06%, respectively. Straw accounted for 3.16% of the changes, that is, the lowest contribution.

**Figure 7:**
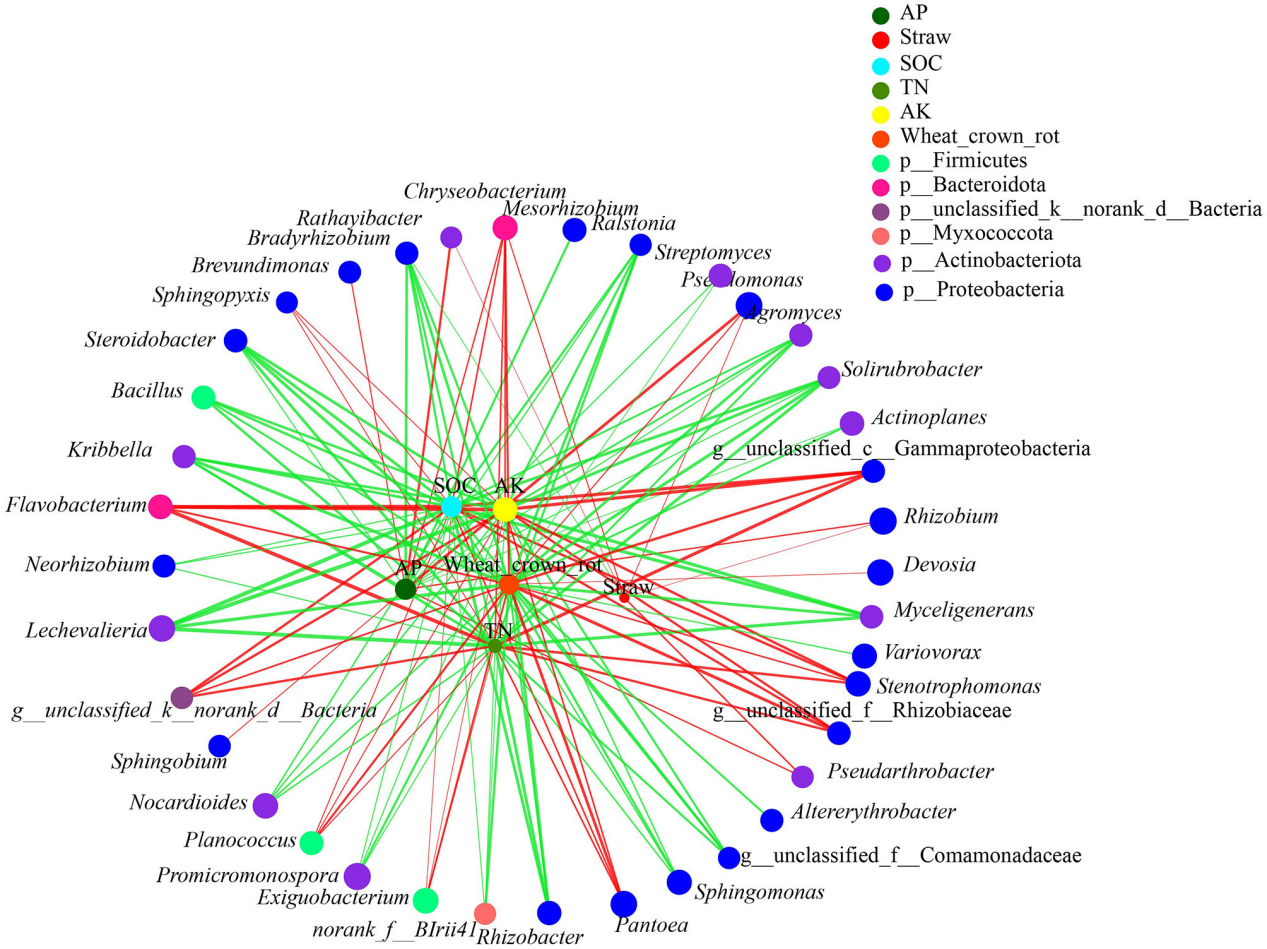
Network analysis of the interaction among endophytic bacterial genera, environmental factors, and wheat crown rot. Red lines, positive correlation; green lines, negative correlation (*p* < 0.05). F1_S, straw returning with no chemical fertilizer application; F2_NS, non-straw returning with moderate chemical fertilizer application; F2_S, straw returning with moderate chemical fertilizer application; F3_NS, non-straw returning with excessive chemical fertilizer application; F3_S, straw returning with excessive chemical fertilizer application; SOC, soil organic carbon; TN, total nitrogen; AK, available potassium, AP, available phosphorus.

The network analysis was used to assess the correlation among the endophytic microbial genera (the 80 most abundant genera), environmental factors, and wheat crown rot (Figure 7). Wheat crown rot positively correlated with 15 genera, among which 13 positively correlated with SOC, TN, and AK, 3 positively correlated with AP, and 2 positively correlated with straw returning. Wheat crown rot negatively correlated with 33 genera, among which 29 positively correlated with SOC, TN, and AK, 12 negatively correlated with AP, and 1 genus was negatively correlated with straw returning.

## 4. Discussion

### 4.1. Effects of straw returning on soil fertility under different fertilization conditions

Crop straws are mainly composed of cellulose, hemicellulose, lignin, protein, and a small number of inorganic salts (Ahmed et al., 2021). After the straw is returned to the field, small molecules of water-soluble substances such as sugars and inorganic substances are first decomposed by bacteria, and then cellulose, lignin, protein, and other macromolecular substances are then broken down by bacteria and fungi. Eventually, the returned straws are degraded by soil microorganisms and release nutrient elements, which supplement the loss of soil fertility (Zhang et al., 2021). In our study, we found that straw returning significantly increased soil fertility under the condition of no fertilization and medium fertilization, which was consistent with the results of previous studies (Wang et al., 2018; Pu et al., 2019). However, we found that, under the condition of excess fertilization, straw returning to the field did not significantly increase soil fertility. The reasons for no significant change may be because of (1) the law of diminishing marginal returns; the law enunciates the principle that the application of successive units of fertilizer to soil gives uniformly decreasing returns if all other factors of production are kept at an optimum. This means that, when the soil has reached high fertility through fertilization, the improvement of fertilization in soil nutrients was lower than that at low fertility (Fippin, 1937; Chen et al., 2021) and (2) poor microbial activity under excess fertilization. The process of straw decomposition by soil microorganisms to release nutrients is influenced by several factors, such as temperature, humidity, soil physical and chemical properties, fertilization, and pesticides (Li et al., 2017; Tang et al., 2021). Excess fertilization can cause soil compaction and acidification, which influence soil microbial community composition and ecological function (Zhao et al., 2019). Furthermore, these changes decreased the soil microbial capacity in crop straw decomposition. Moreover, straw returning under no fertilization altered the function of endophytic bacteria, reduced the abundance of carbohydrate metabolism and nitrogen fixation-related genes, and decreased antibiotic and Lipoarabinomannan synthesis, which may aggravate the occurrence of wheat crown rot. In contrast, straw returning under moderate fertilization had no significant effect on the function of endophytic bacteria. Therefore, considering soil fertility, the function of endophytic bacteria, and the occurrence of wheat stem base rot, fertilizer reduction coupled with straw returning (F2-S) can maintain the soil fertility, increase carbohydrate metabolism and nitrogen fixation, and reduce the occurrence of wheat crown rot.

### 4.2. Fertilizer reduction coupled with straw returning increases the diversity of wheat root endophytes

Endophytes are widely distributed in plants and are influenced by several factors, such as plant species, growth stage, temperature, precipitation, and tillage practices (Papik et al., 2020). In this study, we found that excess fertilization could significantly reduce the diversity of endophytic bacteria, but after fertilizer reduction coupled with straw returning, the richness and diversity of endophytic bacteria increased by 61.20 and 11.93%, respectively. Excess application of fertilizer, especially nitrogen fertilizer, results in the accumulation of nitric acid and ammonia in soil and ultimately reduces soil pH and ammonia toxicity, which reduces the diversity of endophytic bacteria (Geisseler and Scow, 2014). In addition, some rhizospheric bacteria can colonize the surface of the root, then invade the epidermis of the root by active or passive means, and subsequently spread to other parts of the plant (Hardoim et al., 2015). Therefore, the diversity of endophytic bacteria can be influenced by changes in rhizosphere microbial community structure and diversity (Omar and Ismail, 1999). Because straw is rich in microorganisms, straw returning increases soil microbial biomass. Thus, fertilizer reduction coupled with straw returning can increase the diversity of endophytic microbes by increasing soil microbes and reducing the negative impact on soil caused by excessive fertilization.

### 4.3. Excess fertilization and straw returning decreased the nitrogen fixation of endophytic bacteria

Biological nitrogen fixation is the process where the nitrogen-fixing microorganisms reduce inert molecular nitrogen in the atmosphere to ammonia, which is an important part of the nitrogen cycle and an important source of nitrogen absorption by plants (Bahram et al., 2020). Nitrogen-fixing microorganisms can be divided into autophytic, symbiotic, and associative-symbiotic nitrogen-fixing bacteria, based on their relationship with plants. The endophytic bacteria of non-leguminous plants generally assist the plant to absorb nitrogen through associative symbiotic nitrogen fixation (Afzal et al., 2019). The associative symbiotic nitrogen-fixing products of endophytic bacteria have positional advantages such that they are readily absorbed and utilized by plants, instead of soil microorganisms. Endophytic nitrogen-fixing bacteria include *Acetobacter, Burkholderia, Azoarus*, and *Herbaspirillum* (Hecht-Buchholz, 1998; Pedraza, 2008). In our study, *Burkholderia* was the main endophytic nitrogen-fixing bacteria in wheat, and its relative abundance decreased (Supplementary Table S1) with the increase of fertilizer application (F3_NS > F2_NS > F1_NS). Moreover, KEGG analysis showed that the nitrogen fixation capacity of endophytic bacteria decreased with increased fertilization. Thus, our results are consistent with those of previous studies, which suggest that the application of high-nitrogen fertilizer inhibits the colonization of nitrogen-fixing bacteria in plants and reduces their abundance (Salvagiotti et al., 2008). In addition, we found that the nitrogen fixation capacity of endophytic bacteria decreased after the straw returning. However, the relevant mechanisms have not been reported and need further investigation.

### 4.4. Fertilizer reduction coupled with straw returning reduced the occurrence of wheat crown rot

Endophytic bacteria are abundant resources for the biological control of plant diseases. Several endophytic bacteria such as *Bacillus* (Liu et al., 2009; Shu-bin et al., 2012), *Streptomyces* (Shimizu et al., 2009)*, Pseudomonas* (Zhou et al., 2014), and *Burkholderia* (Ho et al., 2015) have been used as biocontrol agents for controlling diseases of several crops. In our study, the relative abundances of *Bacillus, Streptomyces*, and *Burkholderia* decreased with increased fertilization and increased with reduced fertilization coupled with straw returning. In contrast, the relative abundance of *Pseudomonas* decreased with the fertilizer reduction coupled with straw returning, which is mainly related to *Pseudomonascichorii*, that is, a potential pathogen. Therefore, we hypothesized that fertilizer reduction coupled with straw returning may decrease the occurrence of wheat crown rot by increasing the abundance of endophytic biocontrol bacteria.

The possible mechanism of endophytes against plant diseases includes direct and indirect effects. When acting directly, endophytes suppress pathogens by antibiosis. Endophytic bacteria can produce antimicrobial substances to inhibit pathogenic bacteria in the metabolic process, primarily including antibiotics, hydrolases, and alkaloids (Strobel et al., 2004). In the case of indirect effect, endophytic bacteria can trigger ISR to indirectly protect plants against pathogens (Nie et al., 2017; Singh and Gaur, 2017). For numerous resistance-inducing bacterial strains, the ISR-triggering compounds have been identified and shown to comprise the cell wall outer membrane lipopolysaccharide (LPS), iron-chelating siderophores, flagella, antibiotics, etc. (Van Loon, 2007). In our study, antibiotics biosynthesis (penicillin, cephalosporin, carbapenem, neomycin, staurosporine, etc.) and the synthesis of lipopolysaccharide significantly increased with the fertilizer reduction coupled with straw returning. Therefore, we hypothesized that fertilizer reduction coupled with straw returning may increase the occurrence of wheat crown rot by promoting the secretion of antibiotics from endophytic bacteria and inhibiting endophytic bacteria-induced ISR.

## 5. Conclusion

To improve soil fertility and increase food production, the use of excess fertilization is common in China. In our study, we found that excess fertilization reduced the diversity of root endophytic bacteria and the abundance of potential biocontrol bacteria such as *Bacillus, Streptomyces*, and *Burkholderia*, and it inhibited the biosynthesis of antibiotics that aggravate the occurrences of wheat crown rot. Straw returning is an effective measure to improve soil fertility and reduce fertilizer application. This approach not only effectively solves the problem of wasting residue resources but also eliminates challenges related to soil compaction and acidification due to excess fertilization. Our results showed that fertilizer reduction coupled with straw returning maintained the soil fertility, effectively reducing the DNA copies numbers of *F. pseudograminearum* in wheat root and the occurrence of wheat crown rot.

Furthermore, fertilizer reduction coupled with straw returning decreased the community diversity of root endophytic bacteria, changed the structure of the endophytic bacteria community, and increased the relative abundance of potential biocontrol bacteria. Our research provides a theoretical basis for reducing the application of chemical fertilizers and preventing the occurrence of wheat crown rot.

## Data availability statement

The datasets presented in this study can be found in online repositories. The names of the repository/repositories and accession number(s) can be found in the article/Supplementary material.

## Author contributions

YJW: conceptualization, methodology, investigation, and writing the original draft. YXW: disease evaluation and funding acquisition. CYC and XTL: field management and investigation. QSL and WSZ: investigation, bioinformatics, and statistics. SH: sampling campaign and investigation. LXK: conceptualization, formal analysis, validation, reviewing and editing drafts, and supervision. All authors have read and agreed to the published version of the manuscript.
