# Supplementary material for: Effects of fertilizer reduction coupled with straw returning on soil fertility, wheat root endophytic bacteria, and the occurrence of wheat crown rot
Source: Front Microbiol. 2023 Mar 31;14:1143480. doi: 10.3389/fmicb.2023.1143480 (PMC10102530; doi:10.3389/fmicb.2023.1143480)

SUPPLEMENTAL DOCUMENT

Table S1-S4; Figure S1

**Fertilizer reduction** **coupled with** **straw returning decrease the occurrence of wheat crown rot by affecting the endophytic bacteria in wheat root**

Yajiao Wang^1^, Caiyun Cao^2^, Yuxing Wu^1^, Qiusheng Li^1^, Xuetong Liu^2^, Sen Han^1^, Lingxiao Kong^1*^

^1^ Institute of Plant Protection, Hebei Academy of Agricultural and Forestry Sciences, Baoding 071000, China

^2^ Dry Farming Research Institute, Hebei Academy of Agricultural and Forestry Sciences, Hengshui 053000, China

*Correspondence authors: Lingxiao Kong ([konglingxiao163@163.com](mailto:konglingxiao163@163.com))

Table S1 Information of experimental design.

| Treatment | The amount of fertilization | Straw returning |
| --- | --- | --- |
| F1_NS | No fertilization | No |
| F1_S | No fertilization | Yes |
| F2_NS | N fertilizer 180 Kg/ha, P fertilizer 120 Kg/ha,  K fertilizer 120 Kg/ha | No |
| F2_S | N fertilizer 180 Kg/ha, P fertilizer 120 Kg/ha,  K fertilizer 120 Kg/ha | Yes |
| 3_NS | N fertilizer 180 Kg/ha, P fertilizer 120 Kg/ha, K fertilizer 120 Kg/ha | No |
| F3_S | N fertilizer 180 Kg/ha, P fertilizer 120 Kg/ha, K fertilizer 120 Kg/ha | Yes |

Table S2 Information about sequencing.

| samples | Sequence number | Base number | Mean length | OTU | coverage |
| --- | --- | --- | --- | --- | --- |
| F1_NS | 61980.25 | 23383748 | 377.27 | 1119 | 98.72 |
| F1_S | 59467 | 22417663 | 376.96 | 1075 | 98.71 |
| F2_NS | 65841.5 | 24855794 | 377.51 | 1147 | 98.67 |
| F2_S | 62088 | 23421858 | 377.24 | 1190 | 98.55 |
| F3_NS | 63152.5 | 23833928 | 377.41 | 802 | 99.08 |
| F3_S | 67337 | 25403185 | 377.25 | 484 | 99.14 |

F1_NS: non-straw returning with no chemical fertilizer application; F1_S: straw returning with no chemical fertilizer application; F2_NS: non-straw returning with moderate chemical fertilizer application; F2_S: straw returning with moderate chemical fertilizer application; F3_NS: non-straw returning with excessive chemical fertilizer application; F3_S: straw returning with excessive chemical fertilizer application.

Table S3 [Percentage distribution](../../../../C:/Program%20Files%20(x86)/Youdao/Dict/8.9.3.0/resultui/html/index.html#/javascript:;) of dominant genera of endophytic bacteria in wheat root after fertilizer application and straw returning.

| Genera | F1_NS | F1_S | F2_NS | F2_S | F3_NS | F1_S |
| --- | --- | --- | --- | --- | --- | --- |
| *Actinoplanes* | 1.82±0.62 | 0.91±0.47 | 2.38±0.78 | 1.89±0.50 | 0.93±0.27 | 0.41±0.05 |
| *Agromyces* | 1.06±0.53 | 0.47±0.17 | 0.96±0.41 | 0.93±0.49 | 0.16±0.06 | 0.08±0.02 |
| *Allorhizobium* | 6.82±0.98 | 13.42±0.6 | 6.37±0.73 | 8.41±1.75 | 8.33±2.8 | 7.38±0.69 |
| *Altererythrobacter* | 0.60±0.07 | 0.44±0.08 | 0.92±0.19 | 1.05±0.43 | 0.20±0.05 | 0.31±0.05 |
| *Bacillus* | 4.81±1.53 | 0.26±0.11 | 0.38±0.30 | 0.21±0.13 | 0.01±0.00 | 0.09±0.03 |
| *Bradyrhizobium* | 1.51±0.26 | 0.39±0.08 | 0.69±0.09 | 0.59±0.15 | 0.39±0.05 | 0.24±0.03 |
| *Chryseobacterium* | 0.59±0.28 | 1.94±1.64 | 0.70±0.19 | 3.51±3.24 | 2.11±0.78 | 3.54±1.09 |
| *Devosia* | 3.75±0.49 | 4.33±0.94 | 3.51±0.56 | 3.46±1.04 | 8.06±1.84 | 4.94±0.86 |
| *Exiguobacterium* | 0.32±0.26 | 0.08±0.06 | 0.06±0.10 | 0.02±0.01 | 7.78±1.71 | 11.4±3.68 |
| *Flavobacterium* | 0.37±0.19 | 0.76±0.39 | 1.17±0.16 | 1.17±0.59 | 1.91±0.89 | 4.38±1.71 |
| *Kribbella* | 1.46±0.34 | 0.46±0.13 | 0.98±0.28 | 0.79±0.03 | 0.10±0.03 | 0.16±0.03 |
| *Lechevalieria* | 9.78±2.75 | 6.20±1.90 | 6.59±1.88 | 4.85±0.68 | 3.16±1.50 | 1.64±0.39 |
| *Mesorhizobium* | 2.27±1.01 | 0.54±0.13 | 1.05±0.33 | 1.03±0.13 | 0.66±0.16 | 0.71±0.05 |
| *Microbacterium* | 1.01±0.57 | 6.52±0.41 | 4.09±2.66 | 2.80±0.72 | 7.10±0.90 | 4.61±2.80 |
| *Myceligenerans* | 1.46±0.81 | 1.33±0.64 | 1.06±0.58 | 0.59±0.23 | 0.01±0.01 | 0.01±0.00 |
| *Nocardioides* | 3.93±1.42 | 3.26±1.15 | 3.88±1.59 | 4.39±1.77 | 0.55±0.19 | 0.72±0.06 |
| *Pantoea* | 0.58±0.50 | 5.67±3.45 | 0.44±0.27 | 0.72±0.58 | 15.06±2.53 | 13.04±2.31 |
| *Phyllobacterium* | 5.30±3.51 | 2.58±2.36 | 5.95±0.98 | 4.85±1.40 | 8.17±3.01 | 4.02±2.41 |
| *Planococcus* | 0.71±0.99 | 0.73±0.52 | 0.24±0.09 | 0.56±0.46 | 1.67±0.25 | 2.16±1.06 |
| *Promicromonospora* | 14.02±8.33 | 6.30±3.44 | 13.41±2.24 | 13.44±4.83 | 0.98±0.43 | 1.84±0.23 |
| *Pseudomonas* | 1.10±1.27 | 10.91±3.53 | 3.23±1.36 | 5.85±3.02 | 8.36±1.98 | 8.41±2.11 |
| *Ralstonia* | 1.61±0.98 | 0.05±0.0 | 0.27±0.03 | 0.08±0.02 | 0.01±0.01 | 0.02±0.01 |
| *Rathayibacter* | 0.02±0.006 | 1.16±0.77 | 0.035±0.01 | 0.22±0.20 | 0.17±0.05 | 0.04±0.01 |
| *Rhizobacter* | 2.58±0.89 | 1.36±0.64 | 1.24±0.36 | 1.19±0.42 | 0.40±0.12 | 0.66±0.18 |
| *Solirubrobacter* | 1.06±0.27 | 0.42±0.16 | 0.69±0.13 | 0.61±0.29 | 0.10±0.03 | 0.09±0.01 |
| *Sphingobium* | 0.07±0.03 | 1.88±1.51 | 0.10±0.03 | 0.18±0.03 | 0.11±0.02 | 0.07±0.02 |
| *Sphingomonas* | 2.86±0.51 | 2.14±0.27 | 2.90±0.64 | 3.13±0.77 | 0.93±0.33 | 0.81±0.10 |
| *Stenotrophomonas* | 0.44±0.58 | 1.91±0.21 | 2.27±1.82 | 1.45±0.69 | 3.76±1.13 | 5.02±0.70 |
| *Steroidobacter* | 2.17±0.32 | 0.39±0.08 | 1.14±0.27 | 0.84±0.31 | 0.07±0.02 | 0.12±0.02 |
| *Streptomyces* | 1.66±0.39 | 0.27±0.14 | 1.61±0.59 | 1.49±0.44 | 0.15±0.06 | 0.51±0.16 |
| Unclassified Gammaproteobacteria | 0.21±0.05 | 0.64±0.32 | 0.52±0.12 | 0.53±0.05 | 0.10±0.22 | 1.29±0.21 |
| Unclassified Rhizobiaceae | 0.24±0.10 | 0.57±0.23 | 0.28±0.12 | 0.36±0.13 | 1.76±0.23 | 1.32±0.41 |
| Unclassified Xanthomonadaceae | 3.22±1.13 | 1.95±0.57 | 8.71±1.78 | 6.17±0.70 | 2.85±0.29 | 6.60±1.26 |
| *Variovorax* | 1.78±0.34 | 1.64±0.47 | 3.64±0.85 | 2.15±0.41 | 1.34±0.36 | 1.55±0.22 |

F1_NS: non-straw returning with no chemical fertilizer application; F1_S: straw returning with no chemical fertilizer application; F2_NS: non-straw returning with moderate chemical fertilizer application; F2_S: straw returning with moderate chemical fertilizer application; F3_NS: non-straw returning with excessive chemical fertilizer application; F3_S: straw returning with excessive chemical fertilizer application.

Table S4 Correlation coefficient between environmental factors and changes of endophytic bacteria in wheat root after fertilizer application and straw returning.

| Environmental factors | Redundancy analysis | | | | variance partition analysis |
| --- | --- | --- | --- | --- | --- |
|  | RDA1 | RDA2 | r^2^ | p_values | AdjR^2^ |
| SOC | 0.9565 | -0.2919 | 0.5141 | 0.001 | 0.2451 |
| TN | 0.919 | -0.3943 | 0.6312 | 0.001 | 0.2893 |
| AP | 0.8532 | 0.5216 | 0.5637 | 0.002 | 0.2188 |
| AK | 0.9187 | -0.395 | 0.4624 | 0.003 | 0.2006 |
| Straw | 0.5483 | 0.8362 | 0.1874 | 0.127 | 0.0316 |

Figure S1 Function relative abundance of lipoarabinomannan biosynthesis of endophytic bacteria in wheat root after fertilizer application and straw returning. Data for the same index with different letters above bars differed significantly (*p* < 0.05) according to Tukey’s test. F1_S: straw returning with no chemical fertilizer application; F2_NS: non-straw returning with moderate chemical fertilizer application; F2_S: straw returning with moderate chemical fertilizer application; F3_NS: non-straw returning with excessive chemical fertilizer application; F3_S: straw returning with excessive chemical fertilizer application.


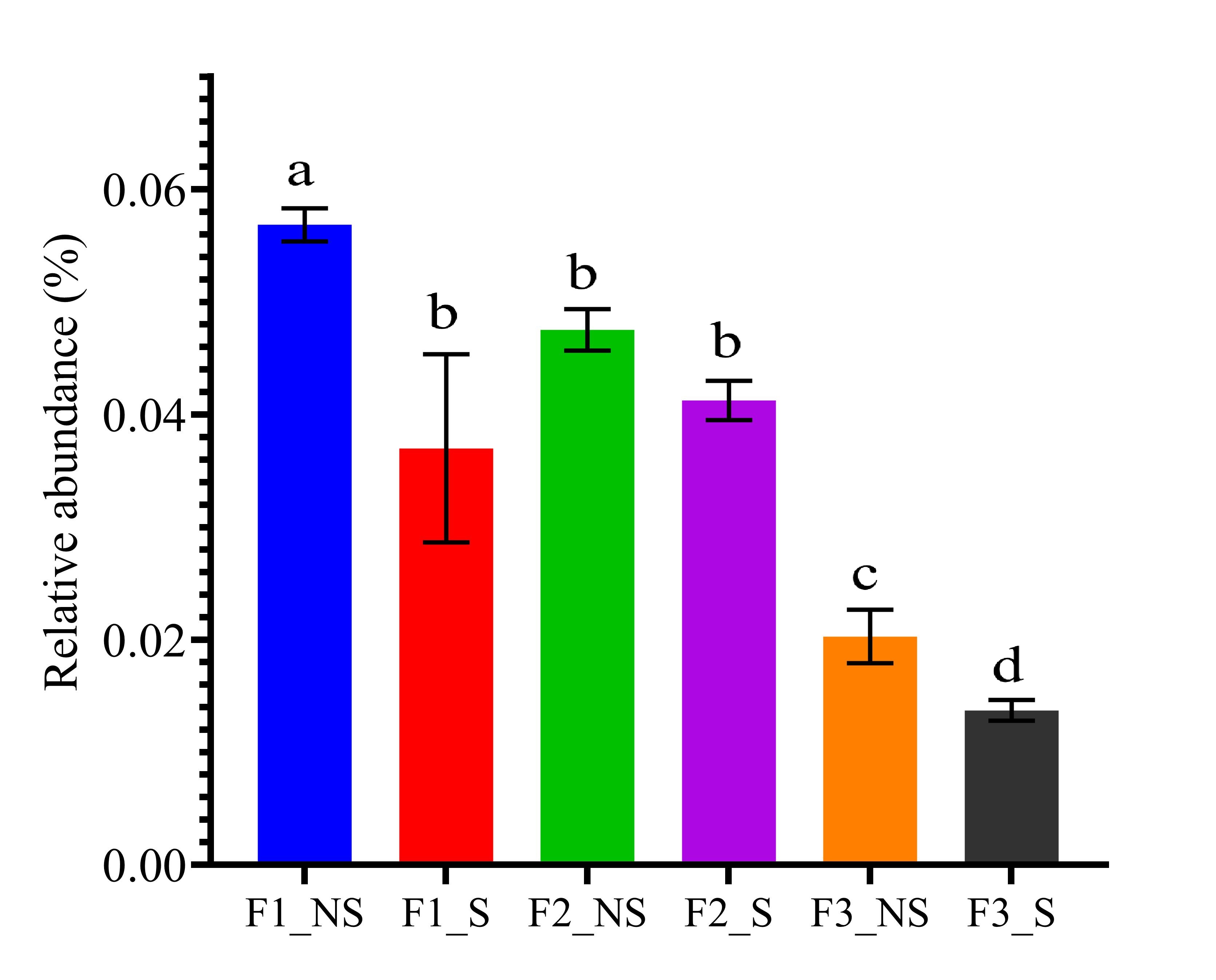

Supplement: Supplementary file 1 [file Table_1.DOC]
